# Human Biodistribution and Dosimetry of ^11^C-CUMI-101, an Agonist Radioligand for Serotonin-1A Receptors in Brain

**DOI:** 10.1371/journal.pone.0025309

**Published:** 2011-09-27

**Authors:** Christina S. Hines, Jeih-San Liow, Paolo Zanotti-Fregonara, Jussi Hirvonen, Cheryl Morse, Victor W. Pike, Robert B. Innis

**Affiliations:** Molecular Imaging Branch, National Institute of Mental Health, Bethesda, Maryland, United States of America; University of Texas, M.D. Anderson Cancer Center, United States of America

## Abstract

As a reported agonist,^11^C-CUMI-101 is believed to selectively bind the G-protein-coupled state of the serotonin-1A (5-HT_1A_) receptor, thereby providing a measure of the active subset of all 5-HT_1A_ receptors in brain. Although ^11^C-CUMI-101 has been successfully used to quantify 5-HT_1A_ receptors in human and monkey brain, its radiation exposure has not previously been reported. The purpose of this study was to calculate the radiation exposure to organs of the body based on serial whole-body imaging with positron emission tomography (PET) in human subjects.

**Methods:**

Nine healthy volunteers were injected with 428±84 MBq (mean ± SD) ^11^C-CUMI-101 and then imaged with a PET-only device for two hours from head to mid-thigh. Eleven source organs (brain, heart, liver, pancreas, stomach, spleen, lungs, kidneys, lumbar spine L1-5, thyroid, and urinary bladder) were identified on whole body images and used to calculate radiation doses using the software program OLINDA/EXM 1.1. To confirm that we had correctly identified the pancreas, a tenth subject was imaged on a PET/CT device.

**Results:**

Brain had high uptake (∼11% of injected activity (IA)) at 10 min. Although liver had the highest uptake (∼35% IA at 120 min), excretion of this activity was not visible in gall bladder or intestine during the scanning session. Organs which received the highest doses (microSv/MBq) were pancreas (32.0), liver (18.4), and spleen (14.5). The effective dose of ^11^C-CUMI-101 was 5.3±0.5 microSv/MBq.

**Conclusion:**

The peak brain uptake (∼11% IA) of ^11^C-CUMI-101 is the highest among more than twenty ^11^C-labeled ligands reported in the literature and provides good counting statistics from relatively low injected activities. Similar to that of other ^11^C-labeled ligands for brain imaging, the effective dose of ^11^C-CUMI-101 is 5.3±0.5 microSv/MBq, a value that can now be used to estimate the radiation risks in future research studies.

## Introduction

For G-protein-coupled receptors, antagonists generally bind with equal affinity to the coupled and uncoupled states, but agonists bind preferentially to the coupled, or active, state of the receptor [1]. Thus, agonist radioligands have the advantage of selectively measuring the subset of receptors that are in the active state [2]. For example, agonist radioligands for the dopamine D_2_ receptor have been used to assess the effects of stimulant-induced dopamine release [3] and agonist-induced internalization of the receptor [4]. The first agonist radioligand introduced for studying a serotonin receptor in human brain with PET (positron emission tomography) was CUMI-101: [*O-methyl*-^11^C]2-(4-(4-(2-methoxyphenyl)piperazine-1-yl)butyl)-4-methyl-1,2,4-triazine-3,5(2*H*,4*H*)dione) [5]. CUMI-101 is a partial agonist at 5-HT_1A_ receptors based on its actions on adenylate cyclase and its action on binding of [^35^S]GTPγS binding to cultured cells with the human receptor [5]. CUMI-101 is reported to be selective for 5-HT_1A_ receptor with an inhibition constant (*K*_i_) of 0.15 nM [5]. In comparison, CUMI-101 binds with much lower affinity to other receptors, such as alpha_1_ (*Ki* = 6.75 nM) and 5-HT_7_ (*Ki* = 12.9 nM), which correspond to 45 and 86 times lower affinity, respectively, than for the 5-HT_1A_ receptor. The uptake of ^11^C-CUMI-101 in monkey brain matches the distribution of 5-HT_1A_ receptors and can be displaced by 5-HT_1A_ receptor-specific ligands [5], [6]. Finally, the uptake of ^11^C-CUMI-101 in human brain has recently been quantified relative to serial concentrations of the radioligand in arterial plasma using compartmental modeling [7], [8]

Based on these recently published studies, ^11^C-CUMI-101 is a promising agonist radioligand to quantify the active state of 5-HT_1A_ receptors in neuropsychiatric disorders. However, the dosimetry of ^11^C-CUMI-101 has not been reported, and this information is typically required for research studies in human subjects. The purpose of this study was to calculate the radiation exposure of ^11^C-CUMI-101 to organs of the body based on whole-body imaging in healthy volunteers.

## Materials and Methods

### Radiopharmaceutical Preparation

^11^C-CUMI-101 was synthesized as described in our Investigational New Drug Application #107,916 (http://pdsp.med.unc.edu/snidd/). In brief, *O*-desmethyl-CUMI-101 (Alpha Biopharmaceuticals, Burlingame, Pennsylvania, USA) was treated with ^11^C-iodomethane. The generated [^11^C]CUMI-101 was separated with reverse phase high-performance liquid chromatography and obtained with a radiochemical purity greater than 99%. Specific activity at time of injection was 86.4±24.1 GBq/micromol.

### Human Subjects

We obtained verbal and written informed consent from all subjects in this study.

The participants in this study were nine healthy volunteers: five males and four females, age 36±12.3 years, weight 89.5±16.5 kg, and BMI 29.6±3.6 (mean ± SD). All subjects were medically and psychiatrically healthy, based on history, Structured Clinical Interview for DSM Disorders – Non-patient, physical examination, EKG, urine toxicology, urinalysis, and blood testing (complete metabolic profile, complete blood count with differential, thyroid stimulating hormone, RPR, HIV, and hepatitis A, B and C antibody testing). The Radiation Safety Committee of the National Institutes of Health and the Combined Neurosciences (CNS) Institutional Review Board of the National Institute of Mental Health approved our ethical use of ^11^C-CUMI-101 in this study of human subjects.

### PET Scan Procedures

We followed the procedure for image acquisition and reconstruction described previously by our group, using GE Advance [9]. In brief, we acquired a 21-min transmission scan (3 min at each of the 7 body segments) using rotating ^68^Ge rods for subsequent attenuation correction. We then administered 428±84 MBq (range 380 to 585 MBq) of ^11^C-CUMI-101 intravenously over 60 seconds using a PHD 2000 syringe pump. We acquired 2D dynamic emission scans consisting of 14 cycles. Acquisition cycles started with an emission scan at the first bed position (i.e., the head) and continued by moving the bed distally for a total of 7 segments. Upon completion of the seventh cycle, the bed was moved back to the original position with the head in the scanner. The 14 cycles increased in duration as follows: 4×0.25, 3×0.5, 3×1, 3×2, and 1×4 min (number of cycles×duration per bed position during that cycle). Movements of the bed between bed positions required 3 s each, and repositioning from thigh back to head at the completion of each of the cycle required 13 s. In total, the emission scan was 120 minutes. All PET images were reconstructed with ordered subset expectation maximization image reconstruction and were corrected for attenuation and scatter. PET images were reconstructed with ordered subset expectation maximization image reconstruction in 28 subsets with four iterations and were corrected for attenuation. All subjects were monitored for safety by checking blood (complete blood count with differential, complete metabolic profile, thyroid stimulating hormone) and urine (urinalysis) within 24 hours before and after the PET scan. Blood pressure and EKG were obtained prior to tracer injection and at 15, 30, and 60 min after injection. To decrease movement during the scan, straps with Velcro adhesive were firmly wrapped around the subject's torso and attached to the bed.

For the PET/CT scan on aSiemens Biograph™ mCT, we obtained a scout image and full body CT (from head to upper thigh) using a frequency of 115 mA. We then administered 369 MBq of ^11^C-CUMI-101 intravenously over 60 seconds using a PHD 2000 syringe pump. We waited 10 minutes to allow radioligand to perfuse the body and bind to receptors in source organs. We then started one cycle of 2D emission scans for each of 7 bed positions, starting with the head. The duration per bed position was 5 minutes long. Thus, we imaged the pancreas at approximately 30 minutes after injection of ^11^C-CUMI-101.

### Additional Monitoring of Human Subjects

Additional monitoring of human subjects was prompted by toxicology studies in rats. For details, see our IND application (http://pdsp.med.unc.edu/snidd/). In brief, rats receiving 1,000 times the human equivalent dose of CUMI-101, had elevated plasma creatinine kinase (CK) in three of ten males and none of ten females. None of the rats with elevated CK died prematurely, and none demonstrated seizures or other physical findings that might be associated with elevated CK. Because of these results, we were required by FDA to monitor subjects with weekly blood draws (CK, MB fraction, troponin I, AST, ALT, and ammonia) for four consecutive weeks after injection of ^11^C-CUMI-101.

### Analysis of PET Images

For each subject, source organs were identified on the individual slices from the PET image averaged over the entire two-hour scanning period using PMOD 3.0 software (PMOD Technologies, Zurich, Switzerland). Brain, salivary glands (parotid and submandibular glands), heart, liver, pancreas, stomach, and spleen were identified in the axial images. Lungs, kidneys, lumbar spine (L1-5), thyroid, and urinary bladder were identified in the coronal direction. Lumbar vertebrae were used to estimate uptake in red marrow, since they contain ∼12.3% of all red marrow in an adult [10]. We used generous-sized regions to encompass all radioactivity from each source organ. Nevertheless, whole body images were viewed with all regions loaded to assure no overlap.

Uptake in each organ was corrected for recovery of measured activity, which was calculated from a large region of interest placed over the entire body for each of the 14 frames. Recovery averaged 90% for all frames. The “remainder of body” was calculated for each time point as the decayed value of the original injected activity minus that present in the identified source organs.

### Residence Time Calculations

At each time point, the measured activity (i.e., not corrected for decay) of the source organ was expressed as a percentage of the injected activity (%IA). The area under the time-activity curve of each organ was calculated by the trapezoidal method during the 120-min scanning session. The area after the last image to infinity was calculated by assuming that the subsequent decline of radioactivity occurred by only physical decay, without any further biological clearance. The area under the curve of %IA from time zero to infinity equals residence time of the organ. The residence time for the lumbar vertebrae was multiplied by a weighting factor (8.13 = 100% / 12.3%) to represent total red marrow in the body. To calculate the residence time for remainder of body, the residence times for all source organs were summed and subtracted from the fixed theoretical value of T_1/2_/ln 2 = 0.49 h.

The software program OLINDA/EXM 1.1 [11] was used to calculate radiation equivalent doses according to the Medical Internal Radiation Dose scheme, using the 70-kg adult male model. Because OLINDA/EXM 1.1 excludes salivary glands as a source organ, we included activity in salivary glands to remainder of body.

## Results

### Pharmacological Effects

Subjects received an injected chemical dose of 5.0±1.0 nmol ^11^C-CUMI-101. This dose caused no pharmacological effects in any subject during the two-hour PET scan, based on patient reports, blood pressure, pulse, temperature, respiratory rate, and EKG.

Because 1,000 times the human equivalent dose of nonradioactive CUMI-101 caused elevated plasma CK in some rats (see Methods), the FDA required us to monitor subjects once per week for four weeks after the PET scan. All subjects were interviewed in person, and blood was drawn to measure CK, CKMB, Troponin I, AST, ALT, and ammonia. The plasma CK was transiently elevated in three of the nine subjects at one to four weeks after injection of ^11^C-CUMI-101. Although these three subjects reported no symptoms, the peak elevations were high: about 900, 1,800, and 2,000 mg/dL, with the normal range being 38–386 mg/dL. After consultations with internists and cardiologists, we interpret theses changes in plasma CK as clinically non-significant, not related to the injection of ^11^C-CUMI-101, but likely caused by vigorous exercise, dehydration, and/or blunt trauma to muscles.

For four weeks after the injection of ^11^C-CUMI-101, all troponin I values were normal and CKMB was never greater than 5% of total CK. Ammonia was mildly elevated in 15% of blood draws but was never higher than 52 micromol/L. Further, the mild elevations in ammonia may have occurred secondary to suboptimal handling of the specimens, which should remain on ice for valid measurements. AST was normal throughout all draws. ALT was elevated once in one subject at the 2 week mark, but to only 5% above the normal upper limit, and this subject reported no complaints.

### Biodistribution

**Figure 1:**
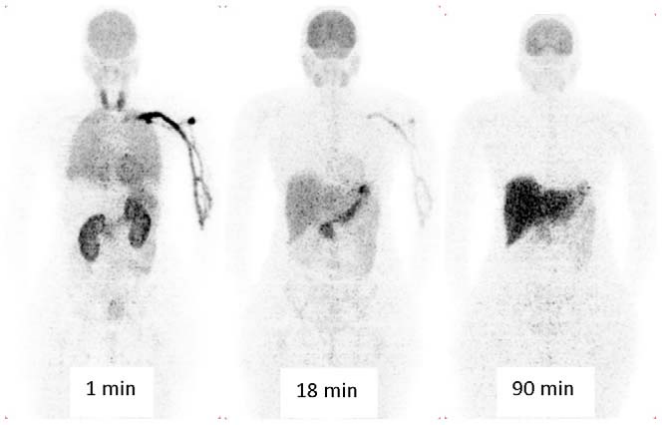
Biodistribution of radioactivity in a female healthy volunteer after injecting ^11^C-CUMI-101 (585 MBq). The high activity at 18 min in the upper left quadrant and adjacent to the liver was thought to be stomach. These planar images are compressed in the coronal direction at the specified times. The right side of the person is on the left side of the image.

**Figure 2:**
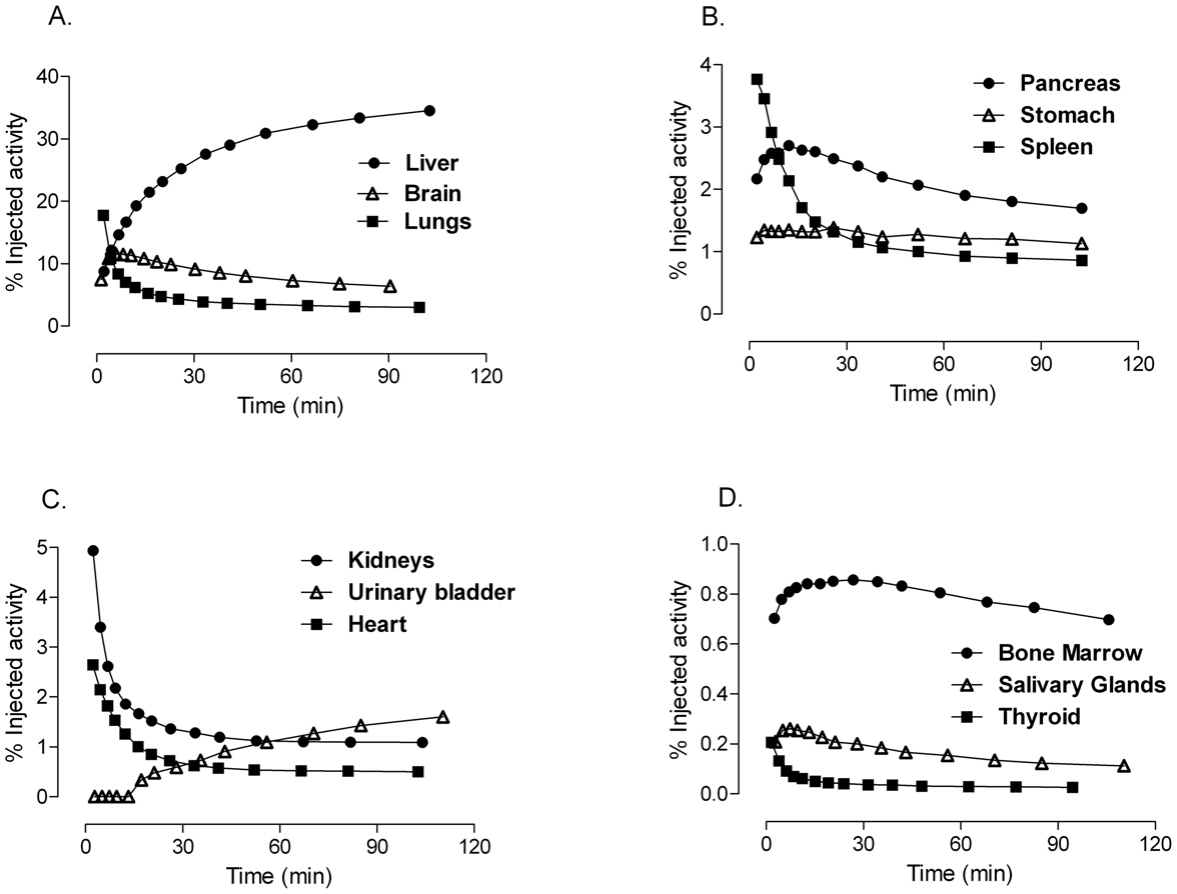
Time-activity curves for identifiable organs after injection of ^11^C-CUMI-101. **A**: Liver (•), Brain (▵), and Lungs (▪). **B**: Pancreas (•), Stomach (▵), and Spleen (▪). **C**: Kidneys (•), Urinary Bladder (▵), and Heart (▪). D: Bone Marrow (•), Salivary Glands (▵), and Thyroid (▪). Data are the average determined in nine healthy volunteers and are corrected for radioactive decay. SD bars are not included to avoid cluttering the graph, but coefficient of variance is reported for residence time, which reflects the area under these time-activity curves (Table 2).

The whole body images were notable for early distribution in the blood pool, accumulation in the target organ (i.e., brain), and apparent metabolism in the liver (Fig. 1). At early time points, radioactivity was prominent in organs with high blood volume, including kidneys, heart, lung, and thyroid (Figure 2). Consistent with its high density of 5-HT_1A_ receptors, the brain had high uptake of radioactivity, with a peak of about 11% IA at 10 min after injection of ^11^C-CUMI-101. This peak brain uptake of 11% IA is high relative to more than 20 other radioligands used for brain imaging: 4.5±3.0, with range of 0.03 to 11 (Table S1). Liver continuously accumulated radioactivity and reached about 35% IA by the end of the scan. Although the high uptake in liver likely represented radiometabolites, we saw no evidence of hepatobiliary excretion during the two-hour scanning period. That is, we saw no significant radioactivity in gall bladder or small intestine. Furthermore, we saw no evidence of significant urinary excretion, since the urinary bladder had on average less than 2% IA at two hours. Because we could not identify the routes of excreting radioactivity, we applied neither the gastrointestinal nor the dynamic urinary bladder models to calculate dosimetry.

**Figure 3:**
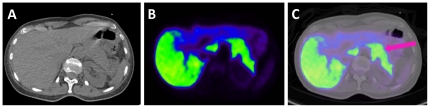
PET-CT of abdomen at approximately 30 minutes after injection of ^11^C-CUMI-101 (369 MBq) shows high uptake in pancreas. **A**: Axial CT of abdomen, with right side of subject on left, and anterior aspect facing upward on page. **B**: PET image of same cross-section, showing high uptake of ^11^C-CUMI-101 in pancreas and liver. **C**: Fused PET-CT image confirms that radioactive signal on the left is at anatomical location of pancreas (pink arrow).

A PET/CT scan in a tenth subject confirmed that we had correctly identified the pancreas as an abdominal organ with relatively high uptake of radioactivity (Fig. 3).

### Dosimetry

If all ^11^C radioactivity remains within the body (i.e., none is excreted), the total residence time equals 0.49 h. Of this total residence time, we attributed about half to eleven source organs and half to remainder of body (Table 1). The mean effective dose derived from nine healthy volunteers was 5.3±0.5 microSv/MBq (19.5±2.2 mrem/mCi). This effective dose (microSv/MBq) is similar to that of 33 other ^11^C-radioligands used for brain imaging, where the mean effective dose was 5.5±2.0, with range of 3.0 to 16.0 (Table S1). Thus, injection of 370 MBq ^11^C-CUMI-101 for a brain study would cause an effective dose of 2.0±0.2 mSv (0.20±0.02 rem). The three organs that received the highest radiation exposure were: pancreas 32.0±12.1 microSv/MBq, liver 18.4±3.1 µSv/MBq, and spleen 14.5±6.9 microSv/MBq.

**Table 1:** Residence times of source organs and remainder of body determined in nine healthy volunteers.

Organ	Residence Time (h)Mean ± SD	COV (%)
Liver	0.1044±0.0189	18%
Brain	0.0442±0.0094	21%
Red marrow	0.0305±0.0088	29%
Lungs	0.0285±0.0191	67%
Pancreas	0.0110±0.0046	42%
Kidneys	0.0089±0.0021	24%
Spleen	0.0088±0.0047	54%
Stomach	0.0060±0.0035	58%
Heart wall	0.0052±0.0018	36%
Urinary bladder	0.0022±0.0030	134%^B^
Salivary glands	0.0009±0.0005	52%^C^
Thyroid	0.0003±0.0001	43%
Remainder of body	0.2405±0.0177	7%
Total^A^	0.4900	

A If all radioactivity remains in the body, the sum of all residence times equals 0.49 h for ^11^C.

B Although this variance was high as a percentage, the mean residence time of the urinary bladder was itself quite small. The small residence time corresponded to accumulation of urinary activity of <2% IA by end of scan (Fig. 2).

C Salivary glands included parotid and submandibular glands. We list the residence time here for comparison with source organs. However, because OLINDA/EXM1.1 excludes salivary glands as a source organ, we attributed activity from salivary glands to remainder of body.

**Table 2:** Radiation dose estimates for ^11^C-CUMI-101 in nine healthy volunteers.

Organ	Dose(µSv/MBq)	SD	%COV
Adrenals	3.71	0.12	3.3%
Brain	10.39	2.14	20.6%
Breasts	1.79	0.08	4.4%
Gallbladder	3.65	0.33	9.0%
LLI	1.27	0.07	5.3%
Small	1.61	0.05	3.2%
Stomach	2.58	0.37	14.5%
ULI	1.74	0.06	3.3%
Heart	2.51	0.08	3.3%
Kidneys	9.86	1.86	18.9%
Liver	18.41	3.11	16.9%
Lungs	8.60	4.79	55.7%
Muscle	1.95	0.06	3.1%
Ovaries	2.08	0.16	7.9%
Pancreas	31.98	12.06	37.7%
Red	1.77	0.09	5.1%
Osteogenic	1.57	0.06	4.1%
Skin	1.50	0.07	4.7%
Spleen	14.54	6.93	47.7%
Testes	1.52	0.06	4.1%
Thymus	2.08	0.09	4.5%
Thyroid	4.55	1.68	37.0%
Urinary	1.90	1.56	82.4%
Uterus	2.07	0.11	5.1%
**Effective Dose**	**5.33**	**0.53**	**10.0%**

## Discussion

Because ^11^C-CUMI-101 is a promising agonist radioligand to image 5-HT_1A_ receptors in brain, we calculated the radiation exposure to organs of the body from whole body images acquired in nine healthy volunteers for two hours after injection of the radioligand. As a measure of exposure to the entire body and weighted for sensitivity to radiation damage, the effective dose was 5.3±0.5 microSv/MBq (19.5±2.2 mrem/mCi), which is comparable to other ^11^C-labeled ligands for brain imaging (Table S1). Thus, injection of 370 MBq ^11^C-CUMI-101 would yield an effective dose of 2.0 mSv, which is well below the commonly used limit of 10 mSv for research studies with minor to intermediate levels of risk (ICRP 62).

The biodistribution of radioactivity after injection of ^11^C-CUMI-101 was notable for high uptake in three organs: brain, liver, and pancreas. Maximal uptake in brain was about 11% IA and occurred at 5–10 min post injection.

We expected substantial brain uptake high because of the high affinity of the radioligand (*Ki* = 0.15 nM) and the moderately high density of 5-HT_1A_ receptors in human brain. Nevertheless, we were surprised by the magnitude of peak brain uptake (11% IA), which is the highest among 24 ^11^C-labeled radioligands with reported values of peak brain uptake (Table S1). Since peak brain uptake occurred early (10 min) and at a time of relatively little binding to receptors, we suspect that physicochemical properties of the radioligand (e.g., optimal lipophilicity) and blood flow were significant factors in the high brain uptake of ^11^C-CUMI-101. Among all organs, the liver had the highest uptake of radioactivity, about 35% IA at two hours. Although this radioactivity presumably represented radiometabolites, we saw no evidence of excretion via gall bladder or small intestine during the two hours of scanning. High uptake of radioactivity in liver and brain caused these two organs to have among the highest radiation exposures (µSv/MBq): liver 18.4±3.1 and brain 10.4±2.1. The third organ with unusually high uptake was the pancreas. In fact, we performed a PET/CT scan in a tenth subject to confirm its identity. We do not know why the pancreas had such high uptake, because this organ is not known to have high densities of 5-HT_1A_ receptors. Furthermore, the radioactivity in pancreas could derive not from CUMI-101 but from a metabolite that has affinity for proteins involved in either the endocrine or exocrine functions of the pancreas.

Because some rats in the animal toxicology study developed elevated plasma concentrations of a muscle enzyme (CK), we monitored subjects weekly for four weeks for evidence of muscle damage. Three of the nine subjects had transiently elevated plasma CK at one to four weeks after injection of ^11^C-CUMI-101. These elevations in CK were asymptomatic and did not impair renal function. We think plasma CK was elevated as a result of intense exercise, dehydration, and/or muscle injury. In retrospect, we recommend that plasma CK not be used to monitor toxicity in subjects who regularly exercise, and we question the utility of monitoring subjects for so long (four weeks) after injection of tracer pharmacological dose of a drug.

In conclusion, based on biodistribution studies in healthy volunteers, the effective dose of ^11^C-CUMI-101 is 5.3±0.5 microSv/MBq (19.5±2.2 mrem/mCi), which is comparable to other ^11^C-labeled ligands for brain imaging. This value can now be used to guide the maximal injected activity in future research studies and to advise subjects of the radiation risks associated with ^11^C-CUMI-101.

## Supporting Information

Table S1 **The effective dose for ^11^C-CUMI-101 was 5.3±0.5 microSv/MBq (19.5±2.2 mrem/mCi), which is comparable to other ^11^C-labeled ligands for brain imaging.** Among 33 other ^11^C-radioligands used for brain imaging, the mean effective dose was 5.5±2.0, with range of 3.0 to 16.0. The peak brain uptake for ^11^C-CUMI-101 was 11% injected activity (IA), the highest relative to more than 20 other radioligands used for brain imaging.
(DOCX)
