# Supplementary material for: Human Biodistribution and Dosimetry of 11C-CUMI-101, an Agonist Radioligand for Serotonin-1A Receptors in Brain
Source: PLoS One. 2011 Sep 27;6(9):e25309. doi: 10.1371/journal.pone.0025309 (PMC3181260; doi:10.1371/journal.pone.0025309)
Supplement: Table S1 — The effective dose for 11C-CUMI-101 was 5.3±0.5 microSv/MBq (19.5±2.2 mrem/mCi), which is comparable to other 11C-labeled ligands for brain imaging. Among 33 other 11C-radioligands used for brain imaging, the mean effective dose was 5.5±2.0, with range of 3.0 to 16.0. The peak brain uptake for 11C-CUMI-101 was 11% injected activity (IA), the highest relative to more than 20 other radioligands used for brain imaging. (DOCX) [file pone.0025309.s001.docx]

**Supplemental Table 1.** Peak brain uptake, dose to the brain, and effective dose of several ^11^C-labeled radiopharmaceuticals (listed from lowest to highest effective dose).

| Radiopharmaceutical | Target | Peak Brain Uptake  (%IA) | Brain Dose  (μSv/MBq) | Effective Dose (μSv/MBq) | Reference |
| --- | --- | --- | --- | --- | --- |
| [^11^C]Glucose | Cell metabolism | 5.7 ^a^ | 11.0^b^  12.2^c^ | 3.0^b^  3.8^c^ | Graham 1998 [1] |
| [^11^C]NPA | Dopamine D2/3 receptor | N/A | 5.5 | 3.2 | Laymon 2009 [2] |
| [^11^C]ABP688 | Metabotropic glutamate receptor subtype 5 (mGluR5) | N/A | 3.6 | 3.7 | Treyer 2008 [3] |
| [^11^C]NMB ^d^ | D2-like dopamine receptor | 3.1^a^ | 3.0 | 3.7 | Antenor-Dorsey 2008 [4] |
| Methyl-[^11^C]thymidine | Cancer imaging | N/A | N/A | 3.8 | Thierens 1994 [5] |
| [^11^C]GSK931145 | Glycine Transporter-1 | 1.4 ^a^ | 2.6^b^  3.0^c^ | 4.0^b^  4.9^c^ | Bullich 2010 [6] |
| [^11^C]MeAIB | Amino acid transport | N/A | 1.6 | 4.0 | Tolvanen 2006 [7] |
| [^11^C]PD153035 | Epidermal growth factor receptor | 2.2 ^a^ | 0.39^b^  0.34^c^ | 4.0 ^b^  5.2^c^ | Liu 2009 [8] |
| [^11^C]MP4B | Butyrylcholinesterase | N/A | 4.7 | 4.2 | Virta 2008 [9] |
| [^11^C]BTA-1 | ß-amyloid plaque | 8.0 | 6.7 | 4.3 | Thees 2007 [10] |
| [^11^C]Choline | Tumors and proliferative disorders | N/A | 1.1 | 4.4 | Tolvanen 2010 [11] |
| [^11^C]carfentanil | mu-opiate receptor | N/A | 4.0 | 4.6 | Newberg 2009 [12] |
| [^11^C]MePPEP | Cannabinoid subtype 1 receptor | 7.9 | 8.0 | 4.6 | Terry 2010 [13] |
| [^11^C](*R*)-PK11195 | Translocator protein 18 kDa (TSPO) | N/A | 1.7 | 4.6 | Kumar 2010[14] |
| [^11^C]docetaxel | Cancer imaging | 0.5 | 0.50 | 4.7 | Van der Veldt 2010 [15] |
| [^11^C]PIB | ß-amyloid plaque | N/A | 3.1 | 4.7 | Scheinin 2007[16] |
| [^11^C](*R*)-PK11195 | Translocator protein 18 kDa (TSPO) | 2.8 ^a^ | 2.5 | 4.8^e^  5.1^f^ | Hirvonen 2009[17] |
| [^11^C]MPGA | GABA receptor | N/A | N/A | 4.8 | Santens 1998 [18] |
| [^11^C](*R*)-Rolipram | Phosphodiesterase 4 | 4.0 | 5.2 | 4.8 | Sprague 2008 [19] |
| Radiopharmaceutical | Target | Peak Brain Uptake  (%IA) | Brain Dose  (μSv/MBq) | Effective Dose (μSv/MBq) | Reference |
| [^11^C]Acetate | Cancer imaging | N/A | 2.1 | 4.9 | Seltzer 2004 [20] |
| [^11^C]MeS-IMPY ^d^ | ß-amyloid plaques | 3.2 | 3.2 | 4.9 | Seneca 2007 [21] |
| [^11^C]Flumazenil | GABA_A_ receptor complex | 10.5^i^ | 9.0* | 5.0 | Nugent 2004 [22] |
| [^11^C]Methionine | Cancer imaging | 2.1 | 3.4 ^g^  3.8 ^h^ | 5.2 ^g^  5.0 ^h^ | Deloar 1998 [23] |
| **[^11^C]CUMI-101** | **Serotonin-1A receptor** | **11.0** | **10.4** | **5.3** | **Current study** |
| [^11^C]PIB | Beta-amyloid plaque | 5.5 | 3.9 | 5.3 | O’Keefe 2009 [24] |
| [^11^C]NNC112 | Dopamine D1 receptor | 7.0 | 6.9 | 5.7 | Cropley 2006 [25] |
| [^11^C]DTBZ ^d^ | Vesicular monoamine transporter type 2 | 2.0 ^a^ | 3.6 ^b^  4.2 ^c^ | 6.1 ^b^  7.0 ^c^ | Murthy 2008[26] |
| [^11^C]MNPA ^d^ | Dopamine D(2/3) receptor | 5.0 | 6.4 | 6.4 | Seneca 2008[27] |
| [^11^C]PE2I | Dopamine transporter | 5.1 | 2.1 | 6.4 | Ribeiro 2007 [28] |
| [^11^C]BTA-1 ^d^ | Beta-amyloid plaque | N/A | 1.7^b^  2.0^c^ | 6.5 ^b^  8.3 ^c^ | Parsey 2005 [29] |
| [^11^C]Raclopride | Dopamine D2 receptor | 3.6 ^a^ | 3.4 | 6.3 | Slifstein 2006 [30] |
| [^11^C]Raclopride | Dopamine D2 receptor | 2.6 | 1.5 | 6.7 | Ribeiro 2005 [31] |
| [^11^C]PBR28 | Translocator protein 18 kDa (TSPO) | 5.0 | 4.8 | 6.6 | Brown 2007 [32] |
| [^11^C]Mirtazapine | Central adrenoceptor | 8.4 ^a^ | 7.9 | 6.8 | Marthi 2003 [33] |
| [^11^C]DASB | Serotonin transporter | 4.0 | 5.8 | 7.0 | Lu 2004 [34] |
| [^11^C]N-desmethyl-loperamide | P-gp transporter | 0.03 | 0.8 | 7.8 | Seneca 2009 [35] |
| [^11^C]Harmine ^d^ | Monoamine oxidase A | 1.5 ^a^ | 2.8 ^b^  3.3 ^c^ | 8.9 ^b^  10.0 ^c^ | Murthy 2007 [36] |
| [^11^C]WAY-100635 | Serotonin 1A receptor | N/A | 2.9 ^b^  4.5 ^c^ | 12.2 ^b^  16.0 ^c^ | Parsey 2005 [37] |
| **Mean ± SD** |  | **4.5±3.0** | **4.3±2.8** | **5.5±2.0** |  |
| **Range** |  | **0.03-11** | **0.34-12.2** | **3.0-16.0** |  |
| **Number radioligands** |  | **24** | **32** | **34** |  |

^a^ Approximate value derived from graph and/or standardized uptake values (SUV) reported in paper. For the conversion in %ID values, we considered an average brain mass of 1,420 g (as specified in the OLINDA software) and the mean weight of the population of subjects used in the study.

^b^ Male

^c^ Female

^d^ Absorbed doses extrapolated from nonhuman primates

^e^ According to ICRP 60

^f^ According to ICRP 103

^g^ Caucasian

^h^ Japanese

^i^Personal communication from Drs Allison Nugent and Peter Herscovitch (NIH)

**References**

1. Graham MM, Peterson LM, Muzi M, Graham BB, Spence AM, et al. (1998) 1-[Carbon-11]-glucose radiation dosimetry and distribution in human imaging studies. J Nucl Med 39: 1805-1810.

2. Laymon CM, Mason NS, Frankle WG, Carney JP, Lopresti BJ, et al. (2009) Human biodistribution and dosimetry of the D2/3 agonist 11C-N-propylnorapomorphine (^11^C-NPA) determined from PET. J Nucl Med 50: 814-817.

3. Treyer V, Streffer J, Ametamey SM, Bettio A, Blauenstein P, et al. (2008) Radiation dosimetry and biodistribution of ^11^C-ABP688 measured in healthy volunteers. Eur J Nucl Med Mol Imaging 35: 766-770.

4. Antenor-Dorsey JA, Laforest R, Moerlein SM, Videen TO, Perlmutter JS (2008) Radiation dosimetry of N-([^11^C]methyl)benperidol as determined by whole-body PET imaging of primates. Eur J Nucl Med Mol Imaging 35: 771-778.

5. Thierens H, van Eijkeren M, Goethals P (1994) Biokinetics and dosimetry for [methyl-^11^C]thymidine. Br J Radiol 67: 292-295.

6. Bullich S, Slifstein M, Passchier J, Murthy NV, Kegeles LS, et al. (2010) Biodistribution and Radiation Dosimetry of the Glycine Transporter-1 Ligand (^11^)C-GSK931145 Determined from Primate and Human Whole-Body PET. Mol Imaging Biol.

7. Tolvanen T, Nagren K, Yu M, Sutinen E, Havu-Auren K, et al. (2006) Human radiation dosimetry of [^11^C]MeAIB, a new tracer for imaging of system A amino acid transport. Eur J Nucl Med Mol Imaging 33: 1178-1184.

8. Liu N, Li M, Li X, Meng X, Yang G, et al. (2009) PET-based biodistribution and radiation dosimetry of epidermal growth factor receptor-selective tracer ^11^C-PD153035 in humans. J Nucl Med 50: 303-308.

9. Virta JR, Tolvanen T, Nagren K, Bruck A, Roivainen A, et al. (2008) 1-^11^C-methyl-4-piperidinyl-N-butyrate radiation dosimetry in humans by dynamic organ-specific evaluation. J Nucl Med 49: 347-353.

10. Thees S, Neumaier B, Glatting G, Deisenhofer S, von Arnim CA, et al. (2007) Radiation dosimetry and biodistribution of the beta-amyloid plaque imaging tracer ^11^C-BTA-1 in humans. Nuklearmedizin 46: 175-180.

11. Tolvanen T, Yli-Kerttula T, Ujula T, Autio A, Lehikoinen P, et al. (2010) Biodistribution and radiation dosimetry of [(^11^)C]choline: a comparison between rat and human data. Eur J Nucl Med Mol Imaging 37: 874-883.

12. Newberg AB, Ray R, Scheuermann J, Wintering N, Saffer J, et al. (2009) Dosimetry of ^11^C-carfentanil, a micro-opioid receptor imaging agent. Nucl Med Commun 30: 314-318.

13. Terry GE, Hirvonen J, Liow JS, Seneca N, Tauscher JT, et al. (2010) Biodistribution and dosimetry in humans of two inverse agonists to image cannabinoid CB_(1)_ receptors using positron emission tomography. Eur J Nucl Med Mol Imaging.

14. Kumar A, Muzik O, Chugani D, Chakraborty P, Chugani HT (2010) PET-derived biodistribution and dosimetry of the benzodiazepine receptor-binding radioligand (11)C-(R)-PK11195 in children and adults. J Nucl Med 51: 139-144.

15. Van der Veldt AA, Hendrikse NH, Smit EF, Mooijer MP, Rijnders AY, et al. (2010) Biodistribution and radiation dosimetry of ^11^C-labelled docetaxel in cancer patients. Eur J Nucl Med Mol Imaging 37: 1950-1958.

16. Scheinin NM, Tolvanen TK, Wilson IA, Arponen EM, Nagren KA, et al. (2007) Biodistribution and radiation dosimetry of the amyloid imaging agent ^11^C-PIB in humans. J Nucl Med 48: 128-133.

17. Hirvonen J, Roivainen A, Virta J, Helin S, Nagren K, et al. (2010) Human biodistribution and radiation dosimetry of ^11^C-(R)-PK11195, the prototypic PET ligand to image inflammation. Eur J Nucl Med Mol Imaging 37: 606-612.

18. Santens P, De Vos F, Thierens H, Decoo D, Slegers G, et al. (1998) Biodistribution and dosimetry of carbon-11-methoxyprogabidic acid, a possible ligand for GABA-receptors in the brain. J Nucl Med 39: 307-310.

19. Sprague DR, Fujita M, Ryu YH, Liow JS, Pike VW, et al. (2008) Whole-body biodistribution and radiation dosimetry in monkeys and humans of the phosphodiesterase 4 radioligand [(^11^)C](R)-rolipram: comparison of two-dimensional planar, bisected and quadrisected image analyses. Nucl Med Biol 35: 493-500.

20. Seltzer MA, Jahan SA, Sparks R, Stout DB, Satyamurthy N, et al. (2004) Radiation dose estimates in humans for ^(11)^C-acetate whole-body PET. J Nucl Med 45: 1233-1236.

21. Seneca N, Cai L, Liow JS, Zoghbi SS, Gladding RL, et al. (2007) Brain and whole-body imaging in nonhuman primates with [^11^C]MeS-IMPY, a candidate radioligand for beta-amyloid plaques. Nucl Med Biol 34: 681-689.

22. Nugent AC, Neumeister A, Drevets WC, Eckelman WC, Channing MA, et al. (2004) Human biodistribution and dosimetry of the PET benzodiazepine receptor ligand ^11^C-flumazenil. Journal of Nuclear Medicine 45(suppl): 434P.

23. Deloar HM, Fujiwara T, Nakamura T, Itoh M, Imai D, et al. (1998) Estimation of internal absorbed dose of L-[methyl-^11^C]methionine using whole-body positron emission tomography. Eur J Nucl Med 25: 629-633.

24. O'Keefe GJ, Saunder TH, Ng S, Ackerman U, Tochon-Danguy HJ, et al. (2009) Radiation dosimetry of beta-amyloid tracers ^11^C-PiB and 18F-BAY94-9172. J Nucl Med 50: 309-315.

25. Cropley VL, Fujita M, Musachio JL, Hong J, Ghose S, et al. (2006) Whole-body biodistribution and estimation of radiation-absorbed doses of the dopamine D_1_ receptor radioligand ^11^C-NNC 112 in humans. J Nucl Med 47: 100-104.

26. Murthy R, Harris P, Simpson N, Van Heertum R, Leibel R, et al. (2008) Whole body ^[11^C]-dihydrotetrabenazine imaging of baboons: biodistribution and human radiation dosimetry estimates. Eur J Nucl Med Mol Imaging 35: 790-797.

27. Seneca N, Skinbjerg M, Zoghbi SS, Liow JS, Gladding RL, et al. (2008) Kinetic brain analysis and whole-body imaging in monkey of [^11^C]MNPA: a dopamine agonist radioligand. Synapse 62: 700-709.

28. Ribeiro MJ, Ricard M, Lievre MA, Bourgeois S, Emond P, et al. (2007) Whole-body distribution and radiation dosimetry of the dopamine transporter radioligand [(^11^)C]PE2I in healthy volunteers. Nucl Med Biol 34: 465-470.

29. Parsey RV, Belanger MJ, Sullivan GM, Simpson NR, Stabin MG, et al. (2005) Biodistribution and radiation dosimetry of ^11^C-WAY100,635 in humans. J Nucl Med 46: 614-619.

30. Slifstein M, Hwang DR, Martinez D, Ekelund J, Huang Y, et al. (2006) Biodistribution and radiation dosimetry of the dopamine D_2_ ligand ^11^C-raclopride determined from human whole-body PET. J Nucl Med 47: 313-319.

31. Ribeiro MJ, Ricard M, Bourgeois S, Lievre MA, Bottlaender M, et al. (2005) Biodistribution and radiation dosimetry of [11C]raclopride in healthy volunteers. Eur J Nucl Med Mol Imaging 32: 952-958.

32. Brown AK, et., al (2007) Radiation Dosimetry and Biodistribution in Monkey and Man of ^11^C-PBR28: A PET Radioligand to Image Inflammation. J Nucl Med 48: 2072-2079.

33. Marthi K, Hansen SB, Jakobsen S, Bender D, Smith SB, et al. (2003) Biodistribution and radiation dosimetry of [N-methyl-11C]mirtazapine, an antidepressant affecting adrenoceptors. Appl Radiat Isot 59: 175-179.

34. Lu JQ, Ichise M, Liow JS, Ghose S, Vines D, et al. (2004) Biodistribution and radiation dosimetry of the serotonin transporter ligand 11C-DASB determined from human whole-body PET. J Nucl Med 45: 1555-1559.

35. Seneca N, Zoghbi SS, Liow JS, Kreisl W, Herscovitch P, et al. (2009) Human brain imaging and radiation dosimetry of ^11^C-N-desmethyl-loperamide, a PET radiotracer to measure the function of P-glycoprotein. J Nucl Med 50: 807-813.

36. Murthy R, Erlandsson K, Kumar D, Van Heertum R, Mann J, et al. (2007) Biodistribution and radiation dosimetry of 11C-harmine in baboons. Nucl Med Commun 28: 748-754.

37. Parsey RV, et. al. (2005) Regional heterogeneity of 5-HT_1A_ receptors in human cerebellum as assessed by positron emission tomography. Journal of Cereral Blood Flow & Metabolism 25: 785-793.
